# Preclinical models in radiation oncology

**DOI:** 10.1186/1748-717X-7-223

**Published:** 2012-12-27

**Authors:** Jenna Kahn, Philip J Tofilon, Kevin Camphausen

**Affiliations:** 1 Radiation Oncology Branch, National Cancer Institute, National Institutes of Health, 10 Center Drive, Building 10, CRC Rm B2-3561, Bethesda, MD, 20892, USA; 2 Warren Alpert School of Medicine at Brown University, Providence, RI, USA

**Keywords:** Preclinical models, Radiation oncology, Radiosensitizer, Orthotopic xenograft model

## Abstract

As the incidence of cancer continues to rise, the use of radiotherapy has emerged as a leading treatment modality. Preclinical models in radiation oncology are essential tools for cancer research and therapeutics. Various model systems have been used to test radiation therapy, including *in vitro* cell culture assays as well as *in vivo* ectopic and orthotopic xenograft models. This review aims to describe such models, their advantages and disadvantages, particularly as they have been employed in the discovery of molecular targets for tumor radiosensitization. Ultimately, any model system must be judged by its utility in developing more effective cancer therapies, which is in turn dependent on its ability to simulate the biology of tumors as they exist *in situ*. Although every model has its limitations, each has played a significant role in preclinical testing. Continued advances in preclinical models will allow for the identification and application of targets for radiation in the clinic.

## Background

It is estimated that in 2012 that there will be 1.6 million non-skin cancers diagnosed in North America; nearly three fourths of these cancer patients will receive radiation therapy sometime during the course of their illness [1]. Because radiotherapy continues to serve as a primary cancer treatment modality, the development of strategies that improve its efficacy could benefit a significant number of patients. Whereas most improvements in tumor control achieved in the past 30 years of radiation oncology can be attributed to advances in dose delivery technology, further improvements will likely depend on a greater understanding of the biology underlying tumor response to radiation. More specifically, it is generally considered that the delineation of the fundamental mechanisms mediating tumor cell radioresistance will lead to the identification of molecules that can serve as targets for radiosensitization. Such information will provide the basis for the development of clinical protocols combining molecularly targeted agents and radiotherapy.

Towards this end, laboratory studies have implicated more than 70 molecules as potential determinants of tumor cell radiosensitivity, with additional molecules identified every year [2]. Thus, there is no shortage of putative molecular targets for radiosensitization. However, translation of these data into clinically relevant targets for cancer radiotherapy requires more than establishing a causal relationship between a molecule and the radiosensitivity of a given tumor cell line: additional information is required. Clearly, whether the target is selective for the radiosensitization of tumor over normal cells should be determined. An additional and more difficult question, regards cellular context. It is often the case for tumor selective targets that they influence the radiosensitivity of some tumor cells, but not others. Defining the genetic, epigenetic and microenvironmental circumstances, i.e. cellular context, in which a molecule functions as a determinant of radiosensitivity would be of considerable value in the development of successful clinical applications. Establishing causal relationships, tumor selectivity and cellular context in a relevant preclinical setting requires the use of a number of experimental models. The goal of this review is to describe the model systems currently employed in research efforts aimed at developing molecular targets for tumor radiosensitization.

## *In Vitro* culture systems

Cells grown in monolayer cultures provide an experimentally expedient model for cancer research in general and have been used extensively in defining the molecular determinants of radiosensitivity. Cell culture experiments typically comprise the initial step in establishing a causal relationship between a suspected molecular determinant and radiosensitivity as well as the preliminary evaluation of drug/radiation combinations. In this model system radiosensitivity is most rigorously defined using the clonogenic survival assay, which measures the proliferative (clonogenic) capacity of individual cells. In the most commonly used form of this assay, single cell suspensions are seeded into tissue culture plates, allowed to attach and irradiated; colonies are then counted after 10-21 days, depending on the specific cell line [3]. The minimum colony size is typically set at 50 cells, which coincides with 5-6 doublings. The necessity for colony formation, which involves a relatively prolonged time period between irradiation and analysis of cell death (defined as the loss of reproductive potential), is based on the fundamental mechanisms of radiobiology. For most solid tumor cells, as well as for fibroblasts, the primary mode of cell death after radiation exposure is mitotic catastrophe, which can require several cell divisions for maximal expression. Moreover, radiation induces transient growth arrest, which can be misinterpreted as cytotoxicity in short term assays based on cell number. With respect to detecting modifications in radiosensitivity, targeting of molecular determinants can potentially switch the mode of death from mitotic catastrophe to apoptosis or autophagy; each form of death is accounted for in the clonogenic survival assay. Thus, the gold standard for quantifying radiosensitivity and its modification is clonogenic survival analysis.

**Figure 1:**
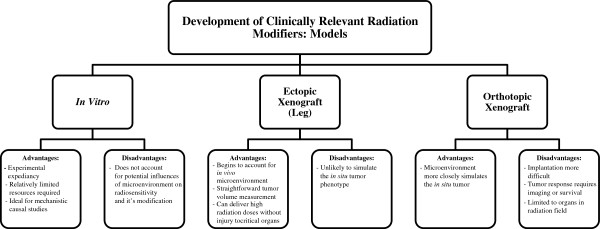
Limitations and advantages of preclinical model systems in radiation oncology.

An additional advantage of cell culture models is the capacity for the molecular manipulation of suspected determinants of radiosensitivity (Figure 1). Standard molecular techniques using genetic and epigenetic approaches have traditionally been combined with the clonogenic survival assay to establish causal relationships between a suspected target and radiosensitization. Furthermore, analyses of such processes as cell cycle phase distribution, DNA DSB induction and repair, and of the mode cell death (apoptosis, mitotic catastrophe or autophagy) provide insight into the mechanism through which the molecule in question affects radioresponse. Cell culture models have also been used in the initial evaluation of whether a molecularly targeted agent acts in a predictable fashion. For example, our lab has previously performed experiments using the HDAC inhibitor valproic acid [4]. Using immunoblots generated from monolayer cultures, valproic acid was shown to induce hyper-acetylation of histone H3 or H4, and this hyperacetylation was dependant on continuous drug exposure. Clonogenic survival analysis then showed that continuous exposure of cells to valproic acid was necessary to achieve the maximum enhancement of radiation induced cell death, consistent with the dependence on continuous drug exposure exhibited by histone hyperacetylation.

Numerous studies aimed at defining the molecular determinants of radiosensitivity have initially been performed using *in vitro* monolayer cultures. Comparison of tumor cell lines to normal cell lines such as fibroblasts or mammary epithelial cells provides for an initial evaluation of whether a molecular determinant and/or a targeted agent selectively enhance the radiosensitivity of tumor cells over normal cells. Using multiple tumor cell lines across various histologies may provide a system for investigating cell the genetic and epigenetic context under which a molecule regulates radiosensitivity.

Although essential to establishing causal relationships between a molecule and radiosensitivity, i.e., identifying molecular targets, the use of *in vitro* model systems is not without limitations. First, the use of *in vitro* systems is based on the assumption that the phenotype of tumor cells in culture recapitulates that of cells in an *in vivo* setting, i.e. the same targets are expressed and are operative. Many commonly used human tumor cell lines have been maintained on plastic in the laboratory for years, allowing for additional unwanted selection pressures; thus, the cell lines may not be representative of the original tumor *in situ*. In a recent study, Shai et al. showed by microarray analysis that tumor cells grown for three passages on plastic were no longer representative of the *in situ* tumors from which they were derived [5]. Furthermore, cells grown *in vitro* lack the architectural and cellular complexity of *in vivo* tumors including inflammatory, vascular, and stromal components [6], factors that can influence tumor radioresponse.

## *In vivo* models

Both rodent and human cells have been used to investigate *in vivo* tumor radioresponse. Most rodent cell studies have focused on implanted murine or rat syngeneic tumors, although more recently genetically engineered mouse (GEM) models have been used to evaluate the radioresponse of spontaneous tumors [7-9]. The primary model for investigating the radioresponse of human tumor cells under *in vivo* conditions involves their implantation and growth in immunocompromised mice or rats [10]. Whereas both rodent and human tumor models are available, studies aimed at developing molecularly targeted radiosensitizing agents have been primarily performed using human tumor cells. The rationale, as recently summarized by Olive and colleagues, is that there are significant differences between rodent and human cells in terms of the fundamental processes regulating radioresponse [11]. Studies performed over the years have shown that the activities and/or levels of critical molecules mediating the DNA damage response after irradiation such as DNA-PK, Ku70, Ku86 and NBS differ between species. Moreover, in terms of general processes, rodent cells are more susceptible to radiation-induced oncogenic transformation, less efficient at checkpoint activation, and more sensitive to oxidative stress as compared to human cells. Given the differences between rodent and human cells regarding aspects of the fundamental mechanisms mediating cellular response and given that a goal of experimental radiation oncology is to develop modifiers relevant to treatment, most preclinical radiation studies focus on human tumor xenografts. However, it should be noted that the human tumor xenograft model does have a number of potentially significant limitations, which include the absence of an immune response and the presence of mouse rather than human stroma. Clearly, depending on the molecular target being addressed and its mechanism of action, these characteristics need to be taken into account when attempting to translate results to a clinical situation.

The most common assay used in defining *in vivo* radioresponse is the tumor growth delay (TGD) assay [3]. Typically, tumor cells, regardless of tissue of origin, are implanted subcutaneously on the leg. This site, in contrast to the flank, allows for delivery of radiation to the tumor without exposing critical organs. After implantation, the tumor is allowed to grow to a measurable volume, the mice are randomized, and therapy is initiated. Tumor volumes are then measured 2-3x weekly with data plotted as the mean of the volumes. However, each tumor should be measured and tracked separately, as the TGD is calculated for each tumor and reported as the difference between the control and experimental group as days delay +/- SEM. Advantages of the leg TGD model include expedient implantation, relatively straightforward volume measurements and minimal impact on the overall wellbeing of the mouse, which limits the introduction of complicating variables. The primary disadvantage is that the microenvironment does not simulate that of the *in situ* tumor.

To account for the potential influence of tumor specific microenvironments, another *in vivo* model used in experimental radiation oncology is the orthotopic implant of tumor cells. This technique has been applied to the radiation response of pancreas, lung, breast, prostate and brain tumors. Our laboratory focuses on brain tumors, as such; we commonly do orthotopic implants of human GBM cells into the brains of nude mice [12]. To perform this technique, a stereotactic apparatus is used to implant tumor cells into a specific area of the brain. Typically, bioluminescent imaging or magnetic resonance imaging can be used to confirm both presence and volume of tumor before initiating the experimental therapy. Irradiation is performed using a customized jig that allows irradiation of the tumor but shields the remainder of the mouse. Once the therapy has begun, the most common endpoint is survival and a Kaplan-Meier curve is generated to determine significance of each therapy. The main advantage of the use of orthotopic tumors is that the microenvironment may more closely represent that of the *in situ* tumor. The disadvantages are the difficultly and skill required to perform the implants, the difficulty of imaging tumors and the impact the tumor implant has on the organ function of the mouse.

## *In vitro vs.* ectopic *vs.* orthotopic models

As described above, each model system used in the development of molecular targets for radiosensitization has advantages and disadvantages. However, the ultimate utility of a given model to the development of more effective cancer therapies depends on its ability to simulate the phenotype of tumor cells as they exist *in situ*. To begin to address this issue, our lab has used gene expression profiling as an indicator of phenotype to compare two human glioma cell lines (U87 and U251) grown *in vitro* as a monolayer and *in vivo* as subcutaneous leg and as intracranial xenograft tumors [13]. For each cell line, the gene expression profile generated from tissue culture was significantly different from that generated from the subcutaneous tumor, which was significantly different from those grown intracranially (i.c.). The U251 and U87 gene expression profiles generated under the three growth conditions were also compared to one another. As expected, the profiles of the two glioma cell lines were significantly different from each other when grown as monolayer cultures. However, the expression profiles for the glioma cell lines were less discordant when grown as subcutaneous tumors, and actually similar when grown as intracranial tumors. Using Statistical Analysis of Microarray software, we also showed that the two cell lines had 290 genes that were common outliers in the orthotopic environment compared to the *in vitro* growth environment (SAM ref). Using the GOStat program, the 290 outlier genes were mapped to functions of central nervous system development and CNS function (GoSTAT ref). These data suggest that the microenvironment has a significant impact on the phenotype of the tumor model and thus the potential response to cytotoxic therapy. This work has been expanded, by additional investigators, to tumors from different *in situ* environments including model systems of ovarian tumors, meduloblastoma, head and neck cancers and lung cancers [14-17].

To determine if the tumor model microenvironment had an effect on the response to radiation therapy we next irradiated U87 and U251 cells as monolayer cultures, as ectopic flank tumors and as orthotopic tumors and collected the tumors for cDNA microarray analysis [18]. The comparison of the arrays for the *in vitro* samples showed very few changes at the mRNA level as well as very few common changes between the cell lines. However, in the samples from the orthotopic tumors there were over 700 common genes that significantly changed after therapy in both cell lines. These data suggest that whereas genotype may be the overwhelming determinant of radiosensitivity for cultured cells, under i.c. conditions the brain microenvironment plays a significant role in regulating the genes affected by radiation. Similar results have been obtained by other investigators using an alternate GBM model as well as an orthotopic pancreas model [19,20]. Thus, taking into account such environmental influences will likely be critical in defining the putative functional significance of radiation-induced changes in gene expression when comparing different model systems.

However, results showing the differences between model systems do not directly address the central question of which model system is most similar to the *in situ* tumors. Currently, there is no biopsy data from patients undergoing radiotherapy after one or several doses of radiation. However, several datasets exist of resected GBM samples studied by microarray profiling [21,22] Shankavaram and colleagues recently completed a comparison of three model systems and the clinical dataset published by Bredel et al. [23]. The data from both the *in vitro* and ectopic *in vivo* samples were not highly correlated with any of the primary GBM samples. However, the data from the orthotopic samples was highly correlated to a subset of the entire clinical database that involved genes and pathways related to neurogenesis and growth factor-induced signal transduction. Moreover, using the combined clinical and experimental datasets, glutamate receptor was suggested as a potential target for radiosensitization unique to the orthotopic system. That is, treatment with the glutamate receptor inhibitor LY341595 was shown to enhance the radiosensitivity of glioma cells grown in the orthotopic model, but not in either the *in vitro* or ectopic tumor models. Thus, these results suggest that the orthotopic tumors represented a subset of the samples collected from patients with GBM and that this subset can be used to discover a novel target for GBM radiosensitization, information that could not be generated from the more standard glioma models of *in vitro* monolayer culture and subcutaneous xenografts.

## Conclusion

The current state of the art in preclinical modeling in radiation oncology research involves the use of *in vitro* cultures and assays, as well as ectopic and orthotopic xenograft models. As investigators initiate pre-clinical experiments, analysis of the genetics of the cell lines used the composition of the microenvironment and the experimental techniques available should be evaluated and compared to the proposed disease to be treated in the clinic.

## Competing interests

The authors declare that they have no competing interests.

## Authors’ contributions

All of the authors participated in the writing, reviewing, and editing of the manuscript. All authors read and approved the final manuscript.
